# Superficial vs. Deep Venous System in DIEP Flaps: Lessons from 25 Years of CTA-Guided Planning

**DOI:** 10.3390/jcm14175972

**Published:** 2025-08-24

**Authors:** Ferruccio Paganini, Sara Matarazzo, Beatrice Corsini, Elvio De Fiori, Andrea Manconi, Luigi Valdatta, Valeria Navach, Cristina Garusi

**Affiliations:** 1 Division of Plastic and Reconstructive Surgery, Department of Biotechnology and Life Sciences, University of Insubria, Via Ravasi, 2, 21100 Varese, Italy; 2 Department of Radiology, European Institute of Oncology (IEO), 20141 Milan, Italy; 3 Department of Senology, ICS Maugeri Pavia, 27100 Pavia, Italy; 4 Division of Plastic Surgery, AUSL Piacenza, 29121 Piacenza, Italy; 5 Department of Plastic Surgery and Burn Unit, Niguarda Ca’ Granda Hospital, 20162 Milan, Italy

**Keywords:** breast, breast reconstruction, autologous, DIEP, tertiary breast reconstruction, complications, outcomes, microsurgery, flaps

## Abstract

**Background**: Venous congestion is a major contributor to complications in DIEP flap breast reconstruction. Beyond superficial venous dominance, the presence or absence of anatomical connections between the superficial and deep venous systems may influence drainage physiology. This study investigates how preoperative CTA and targeted superdrainage impact outcomes over a 25-year period. **Patients and Methods**: A retrospective analysis was conducted on 208 DIEP flaps performed from 2000 to 2024 at a single center. From 2006, computed tomographic angiography (CTA) was routinely used to evaluate venous anatomy, focusing on the presence, trajectory, and connection of the superficial inferior epigastric vein (SIEV) with the deep system. Superdrainage was performed when superficial venous dominance was evident or drainage was judged insufficient intraoperatively. Primary outcomes included venous congestion, partial necrosis, and reoperations; secondary outcomes included hospital stay and safety of superdrainage. **Results**: Venous complications decreased significantly after CTA implementation (37.5% vs. 8.0%; *p* < 0.001). Superdrainage was performed in 40.9% of post-CTA cases, with 90% preoperatively planned based on CTA findings. No complications were associated with second venous anastomosis. Flap outcomes correlated not with perforator number or flap size but with venous drainage physiology. Mean hospital stay was shorter post-CTA (6 vs. 9 days; *p* < 0.001). **Conclusions**: Evaluating the anatomical connection between superficial and deep venous systems via CTA enhances venous planning and allows for safer, physiology-driven decisions. In the absence of such connections, intraoperative evaluation remains essential. Drainage physiology—rather than anatomical metrics alone—should guide surgical strategy in DIEP flap reconstruction.

## 1. Introduction

The deep inferior epigastric perforator (DIEP) flap has become the gold standard in autologous breast reconstruction, offering optimal aesthetic results while minimizing donor-site morbidity [1,2,3,4]. However, despite its many advantages, venous congestion remains a critical complication that can threaten flap viability and compromise surgical outcomes [5,6,7,8]. The venous anatomy of the lower abdominal wall involves two distinct systems: the deep venous system, primarily the deep inferior epigastric vein (DIEV), and the superficial system, mainly the superficial inferior epigastric vein (SIEV) [7,8,9,10]. The balance between these two systems is not fixed—anatomical variations exist, and either system may be dominant in terms of venous outflow.

Even when the SIEV appears to have a good caliber upon dissection and clamping, this does not necessarily indicate that the deep system will be insufficient [7]. In fact, in many cases, the deep venous system via the venae comitantes of the selected perforators is sufficient. Importantly, anatomical studies and intraoperative findings have demonstrated that anastomotic connections exist between the superficial and deep systems, allowing for compensatory drainage when one pathway is insufficient. However, when these connections are inadequate or absent and the dominant system is not properly addressed surgically, venous congestion may ensue.

Historically, the management of venous outflow in DIEP flaps relied heavily on intraoperative assessment and surgeon experience. In the early 2000s, prior to the routine use of preoperative computed tomographic angiography (CTA), perforator selection and venous evaluation were performed without detailed imaging guidance, resulting in a higher incidence of venous complications [3,11,12,13,14].

Since 2005, our center has implemented standardized preoperative CTA protocols, enabling detailed mapping of both arterial perforators and venous drainage pathways [15,16,17,18]. This has allowed for more accurate surgical planning, including recognition of venous dominance and distribution of SIEV as well as anticipation of the need for additional venous anastomoses.

The primary objective of this retrospective study is to evaluate the role of the superficial–deep venous system connection in DIEP flap survival and complication rates, across a 25-year period in a single high-volume center. A secondary aim is to assess the impact of the introduction of preoperative CTA on the incidence of venous complications, comparing outcomes before and after its implementation.

## 2. Patients and Methods

This is a retrospective observational study conducted at a single high-volume tertiary center, including all breast reconstructions performed using the deep inferior epigastric perforator (DIEP) flap technique between January 2000 and December 2024. A total of 208 DIEP flap reconstructions were analyzed.

The study population was divided into two cohorts based on the availability of preoperative imaging:

- Group 1 included all procedures performed before the introduction of preoperative computed tomographic angiography (CTA), between January 2000 and March 2005 (n = 32).
- Group 2 included all procedures performed after the routine implementation of CTA, from April 2005 to August 2024 (n = 176). A standard imaging acquisition protocol was developed internally between March and November 2005 and has remained essentially unchanged since January 2006.

**Figure 1:**
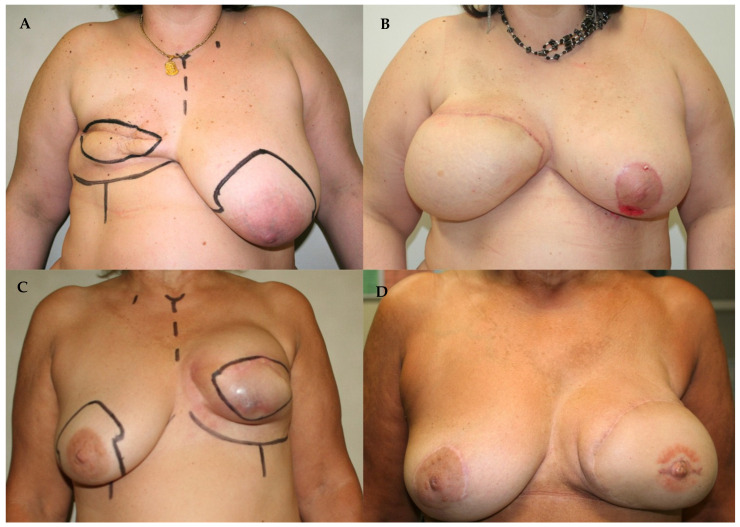
(**A**) Preoperative image of a patient with iatrogenic amastia following right mastectomy. (**B**) Postoperative result at 4 weeks after right breast reconstruction with a free DIEP flap and simultaneous contralateral breast symmetrization by reduction mammaplasty. (**C**) Preoperative image of a patient with Baker grade IV capsular contracture after two-stage implant-based reconstruction following left mastectomy. (**D**) Postoperative result at 12 months after tertiary reconstruction with a DIEP flap and contralateral symmetrization by mastopexy, nipple reconstruction using local C-V flaps, and areolar pigmentation.

**Figure 2:**
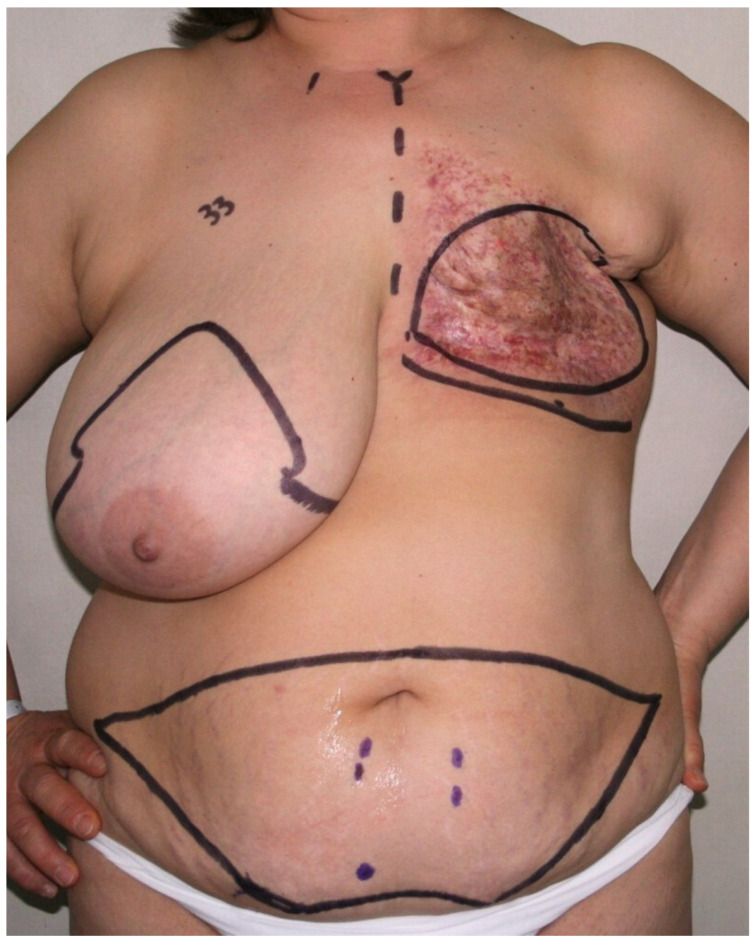
Preoperative markings in a patient with severe radiation dermatitis following adjuvant radiotherapy after left mastectomy. Markings indicate planned left breast reconstruction and contralateral reduction mammaplasty for symmetrization. On the abdomen, the preoperative design of the DIEP flap is shown, highlighting the perforators of the deep inferior epigastric artery, identified using preoperative CTA and handheld Doppler.

All patients undergoing DIEP flap reconstruction, either immediate or delayed, unilateral or bilateral, were eligible for inclusion. A minimum follow-up of 12 months was required for inclusion. The following exclusion criteria were applied: (1) use of alternative flaps (TRAM, SIEA), (2) combined or salvage procedures, (3) missing intraoperative or postoperative outcome data, and (4) complete absence of preoperative demographic data. Patients with isolated missing demographic information were not excluded. Out of 243 total DIEP procedures during the study period, 208 met the inclusion criteria (Figure 1 and Figure 2).

**Figure 3:**
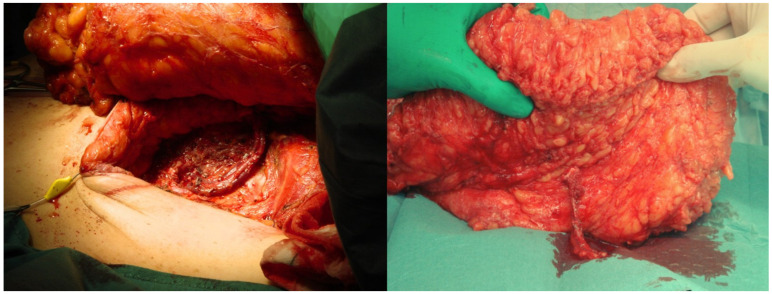
Intraoperative dissection of the DIEP flap pedicle. The pedicle is isolated, and the flap is harvested following pedicle division.

All procedures were performed by the same senior reconstructive surgeon, assisted by a stable operative team composed of two to three additional members. The standard operative technique involved intramuscular perforator dissection under loupe magnification and flap harvest with preservation of the rectus abdominis muscle (Figure 3). All arterial and venous anastomoses were performed in an end-to-end fashion using standard microsurgical suture materials; vascular couplers were never used. The preferred recipient vessels were the internal mammary artery and vein, accessed through the second or third intercostal space. When a second venous anastomosis (i.e., superdrainage) was performed, the most used recipient veins were lateral thoracic veins or superficial collaterals directed toward the axilla or even mastectomy veins. The decision regarding secondary venous drainage was made based on intraoperative clinical judgment or, after 2005, guided by CTA findings. Close evaluation of the flap itself at the end of the dissection and before the ischemia time was crucial, with indocyanine green (ICG) technology being not available.

Flap perfusion and venous drainage were assessed visually and by gentle milking after perforator dissection at the donor site and again at the recipient site after completion of microvascular anastomoses. The intraoperative evaluation was performed approximately 5–10 min after pedicle reperfusion to allow for stabilization of flow.

From 2005 onward, CTA allowed for detailed preoperative planning. The presence of a well-developed superficial venous system was systematically recorded, and the diameter of the superficial inferior epigastric vein (SIEV) was measured when visible. CTA image acquisition was performed using a dedicated protocol developed and refined in-house. Two radiology technicians trained specifically for perforator mapping carried out all scans, in collaboration with the attending radiologist and operating surgeon. Image interpretation and surgical planning were performed primarily by the reconstructive surgeons.

**Figure 4:**
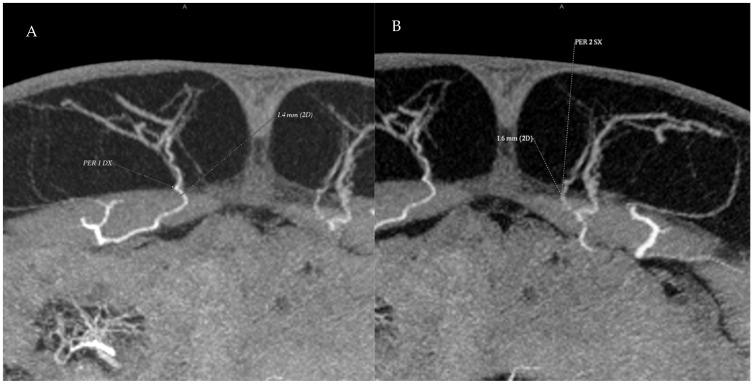
Preoperative CTA images showing the perforating vessels of the deep inferior epigastric artery in the periumbilical region. (**A**) A single right-side perforator with a diameter of 1.4 mm is identified. (**B**) Two adjacent left-side perforators are visible; the more medial perforator measures 1.8 mm in diameter.

**Figure 5:**
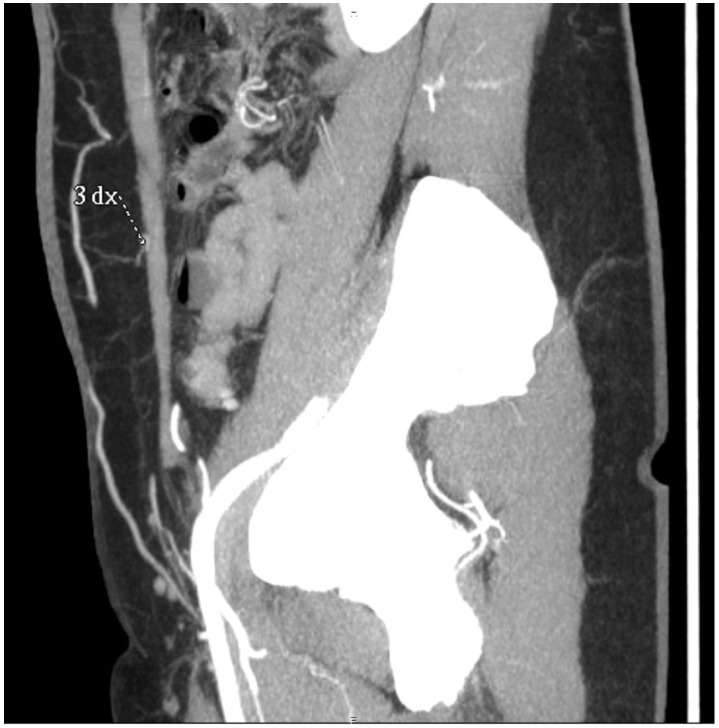
Sagittal sections of preoperative CTAs in the venous phase, showing the superficial venous system. The superficial inferior epigastric vein (SIEV) is clearly visualized.

CTA acquisition was performed in an angiography-like fashion, starting from the moment contrast became visible in the femoral vessels until its opacification reached the umbilical level and periumbilical perforators. The defined pathway of the SIEV, the course of the dominant perforator on each side, its relation to the muscle, its location within the flap, and any visible connection between the SIEV and DIEV at the perforator level were analyzed during the reconstruction of the images of the MIP of the CT scan. Slice thickness in CTA acquisition was 0.625 mm (Figure 4 and Figure 5).

In the final months of 2024, intraoperative fluorescence imaging with indocyanine green (ICG) was introduced for perfusion assessment. However, due to the limited number of cases and insufficient training time, this method was not incorporated into the standard preoperative planning protocol and is not formally analyzed in this study.

Postoperative care followed a standardized protocol. Flap monitoring was performed every 2 h for the first 24 h using clinical parameters (skin color, temperature, capillary refill) and handheld Doppler signal [6,19]. Monitoring continued every 4 h during the second postoperative day, and, from postoperative day 3 onward, clinical evaluation was performed every 8 h. Patients were mobilized on postoperative day 2. All patients received pharmacologic thromboprophylaxis with weight-adjusted subcutaneous enoxaparin and wore graduated compression stockings throughout hospitalization, which lasted a minimum of 5 days.

Considered data were the incidence of venous complications, defined as intraoperative or postoperative venous congestion, partial flap necrosis, or total flap loss. Secondary outcomes included the rate of additional venous anastomoses (superdrainage), need for surgical revision, length of hospital stay, and the incidence of systemic complications such as seromas, infections, or thromboembolic events.

No multivariate analysis was performed due to the retrospective design and the relatively low number of adverse events in the post-CTA cohort, which would limit statistical power and risk overfitting.

Comparative analysis was performed between the pre-CTA and post-CTA groups. Descriptive statistics were used to summarize demographic and clinical variables. Chi-square or Fisher’s exact tests were applied to categorical data, and Student’s *t*-tests were used for continuous variables. A *p*-value < 0.05 was considered statistically significant.

Statistical analysis was performed using IBM SPSS Statistics for Windows, Version 29.0 (IBM Corp., Armonk, NY, USA) and Microsoft Excel for Microsoft 365 (Microsoft Corp., Redmond, WA, USA).

This study was conducted in accordance with the principles of the Declaration of Helsinki (latest revision) and reported in line with the STROBE (Strengthening the Reporting of Observational Studies in Epidemiology) guidelines for retrospective studies [20] (see Supplementary Materials, Table S1).

## 3. Results

### 3.1. Patient Characteristics

A total of 208 patients underwent DIEP flap breast reconstruction and were included in this analysis. A total of 32 procedures (15.4%) were performed before the introduction of computed tomographic angiography (CTA) (pre-CTA group), and 176 (84.6%) were performed after its implementation (post-CTA group). The mean age of the overall cohort was 47.04 ± 8.55 years. Patients in the post-CTA group were significantly older than those in the pre-CTA group (48.58 ± 5.32 vs. 42.35 ± 13.06 years; *p* < 0.001). The mean BMI was 26.21 ± 4.68, with no significant difference between the two groups (27.44 ± 6.43 pre-CTA vs. 26.03 ± 4.32 post-CTA; *p* = ns). All patients had abstained from smoking for at least 30 days prior to surgery.

Hypertension was present in 16 patients (5 pre-CTA, 11 post-CTA), with no significant difference between groups (*p* = 0.14). Diabetes mellitus was significantly more common in the pre-CTA group (9/32, 28.1%) than in the post-CTA group (14/176, 7.95%; *p* = 0.002). Unilateral reconstructions were performed in 188 patients (90.4%) and bilateral in 20 patients (9.6%). These characteristics are summarized in Table 1.

### 3.2. Primary Outcomes

Venous-related complications were significantly more frequent in the pre-CTA group. Specifically, postoperative venous congestion occurred in 12/32 patients (37.5%) pre-CTA and in 14/176 patients (8.0%) post-CTA (*p* < 0.001). Partial flap necrosis was observed in 10 cases (31.2%) pre-CTA and in 13 cases (7.4%) post-CTA (*p* = 0.003). Total flap loss occurred in two patients (6.3%) in the pre-CTA group and in one patient (0.6%) in the post-CTA group (*p* = 0.11, Fisher’s exact test). Re-exploration due to venous insufficiency with secondary venous anastomosis on the first postoperative day was performed in 8 pre-CTA cases (25%) and 12 post-CTA cases (6.8%), with a statistically significant difference (*p* = 0.007). These outcomes are detailed in Table 2.

### 3.3. Secondary Outcomes

Superficial venous drainage (superdrainage) was performed in 3 patients in the pre-CTA group (based on intraoperative findings) and in 72 patients in the post-CTA group. No complications were associated with any superdrainage procedures. No temporal trend was observed in the indication or frequency of superdrainage during the post-CTA period. These data are presented in Table 3.

Mean length of hospital stay was significantly reduced in the post-CTA group compared to the pre-CTA group (6 ± 2 vs. 9 ± 3 days; *p* < 0.001), as shown in Table 4.

In the post-CTA cohort, 72/176 flaps (40.9%) underwent superdrainage. Among these, 65/72 (90.3%) were preoperatively planned on CTA and confirmed intraoperatively, whereas 7/72 (9.7%) were not anticipated on CTA and were performed based on intraoperative evidence of venous congestion. Our database captured only superdrainage procedures that were actually performed; therefore, we could not quantify instances in which CTA suggested superdrainage but it was ultimately omitted intraoperatively due to adequate venous drainage.

Overall, the introduction of preoperative CTA and the systematic planning of superficial venous drainage were associated with a significant reduction in venous complications and hospital stay duration.

## 4. Discussion

This retrospective study demonstrates that the systematic use of preoperative computed tomographic angiography (CTA) over a 25-year period significantly reduced venous complications in DIEP flap breast reconstruction. By evaluating the venous anatomy prior to surgery—particularly the presence, trajectory, and connections of the superficial inferior epigastric vein (SIEV)—the surgical team could plan for a second venous anastomosis when appropriate [7,21,22,23]. This approach resulted in a rational and reproducible use of superdrainage, which was not associated with any morbidity in our experience.

Venous outflow remains one of the most critical elements for the success of DIEP flaps. In our planning protocol, particular attention is paid not only to the presence of a dominant superficial venous system but also to its anatomical relationship with the dominant perforator. Rather than simply measuring venous caliber, we consider it essential to evaluate whether a visible connection exists between the SIEV and the deep system via the perforator. This anatomical linkage—when present—suggests an integrated venous network that can be effectively drained through the deep anastomosis alone, providing optimal physiology. In such cases, flap behavior is often predictable and stable, and superdrainage is rarely required.

Conversely, when no direct connection is observed on CTA, preoperative planning loses part of its predictive value, and intraoperative clinical evaluation becomes central. The quality of drainage is then assessed by observing the flap’s color, turgor, and venous behavior after harvesting. Importantly, the decision to perform a second venous anastomosis in these cases is not based on the number of perforators—always one in our protocol—or on flap volume but solely on the flap’s drainage physiology. This functional approach reflects the real-time hemodynamic response of the tissue and is a core principle of our reconstructive strategy.

**Figure 6:**
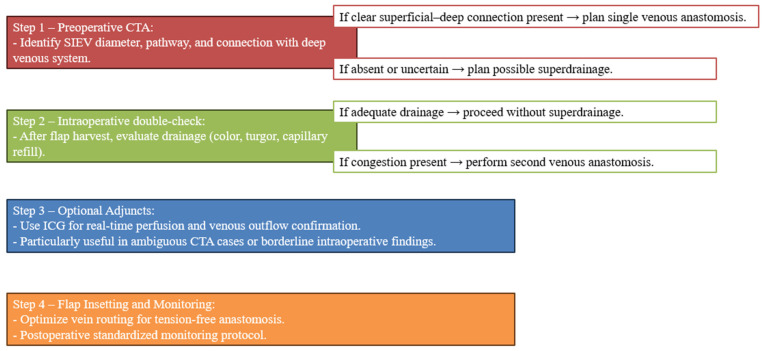
Personalized algorithm for venous outflow management in DIEP flaps based on preoperative CTA, intraoperative assessment, and optional ICG imaging.

Based on our 25-year experience and the integration of CTA findings with intraoperative physiological assessment, we developed a stepwise decision-making algorithm for venous outflow management in DIEP flaps (Figure 6). This algorithm summarizes the key preoperative, intraoperative, and postoperative decision points, including the potential role of ICG imaging, and is intended as a practical guide to support anatomy-driven, patient-specific surgical planning.

These findings align with the previous literature identifying venous congestion as a major cause of flap compromise. While arterial perforator selection has long been a focus in DIEP planning, our data reinforce the importance of systematic venous analysis. Moreover, they support a shift from rigid protocols toward anatomy-driven, patient-specific decision making. Superdrainage should not be interpreted as a failure of planning but as an adaptive technique aligned with individual venous architecture and physiology.

Superdrainage, however, is not entirely without trade-offs. Even when planned, it involves an additional anastomosis and adds a measurable amount of time to the procedure. Although this was not formally recorded in our dataset, the impact on operative workflow should be acknowledged. Furthermore, when a second vein is anticipated, CTA planning allows not only for deciding whether to perform it but also for identifying its precise location. This facilitates a more ergonomic and efficient insetting of the flap, particularly in bilateral or anatomically complex cases.

Another strength of our protocol lies in the fact that CTA images were interpreted directly by the surgical team. This reinforces the importance of surgeon-led imaging analysis in microsurgical planning: reading the scan is not just a diagnostic step but an active part of decision making.

While arterial factors were not the focus of this study, all cases followed a standardized perforator selection protocol based on CTA. We acknowledge that perforator quality may influence outcomes, but these variables were presumed to be balanced across the study periods.

This study evaluated early surgical outcomes such as venous congestion, partial necrosis, and reoperations. Long-term data on aesthetic results and patient-reported outcomes were not included but represent important areas for future research. It is plausible that improved venous physiology also contributes to better long-term volume retention and patient satisfaction.

CTA, despite its advantages, is not without limitations. It involves exposure to radiation and contrast agents, and it requires coordination with radiology, which may delay or complicate planning. Moreover, it provides static anatomical information that does not capture intraoperative dynamics.

Technologies such as indocyanine green (ICG) fluorescence imaging may address some of the limitations of CTA. ICG offers real-time perfusion assessment without radiation exposure or nephrotoxic contrast agents, and its use is entirely surgeon-driven [24]. Introduced in our center only in late 2024, ICG imaging was not systematically evaluated in this study; however, our preliminary experience suggests that it provides rapid and intuitive visualization of both arterial inflow and venous outflow at the bedside. This capability can refine intraoperative decisions regarding the need for superdrainage, particularly in cases where CTA does not reveal a clear superficial–deep venous connection. We foresee a complementary role for these modalities, with CTA offering detailed preoperative anatomical mapping and ICG enabling dynamic intraoperative physiology assessment. In selected settings, especially when rapid intraoperative decision making is critical, ICG may eventually complement or even replace CTA. Alongside high-resolution ultrasound, these tools have the potential to create a more dynamic, flexible, and operator-dependent model of reconstructive planning. They also serve as valuable educational resources for surgical trainees, enhancing intraoperative anatomical understanding and decision-making skills.

This study has several limitations that should be acknowledged. It is a retrospective analysis conducted in a single center, which inevitably restricts the generalizability of its findings. Moreover, we did not include patient-reported outcome measures (PROMs) or long-term aesthetic evaluations, which would have allowed us to correlate venous physiology with patient satisfaction over time. The small number of adverse events in our cohort also precluded a meaningful multivariate analysis. In addition, we recognize that surgical expertise, team coordination, and perioperative care evolved considerably over the 25-year study period, potentially influencing outcomes alongside the introduction of CTA. Our experience with intraoperative ICG fluorescence imaging was limited to the very final months of data collection, preventing us from evaluating its role in a systematic manner. Finally, while CTA provides high-resolution anatomical mapping, it remains a static preoperative assessment and cannot capture the dynamic physiological changes that occur intraoperatively.

Ultimately, the most reliable predictor of success in DIEP flap surgery is not the number of perforators nor the flap volume but the flap’s drainage physiology—whether determined through CTA or assessed intraoperatively. Recognizing and respecting this physiology is the cornerstone of safe and effective microsurgical breast reconstruction.

## 5. Conclusions

In this retrospective 25-year experience, the introduction and standardization of preoperative computed tomographic angiography significantly reduced the rate of venous complications in DIEP flap breast reconstruction. Accurate identification of venous anatomy, particularly superficial system dominance, allowed for tailored surgical planning, including the safe and effective use of superdrainage. These findings reinforce the central role of venous outflow assessment in flap viability and highlight the value of surgeon-led, anatomy-based decision making. While CTA remains a powerful planning tool, future developments in intraoperative imaging—such as ICG fluorescence and high-resolution ultrasound—may offer more dynamic, flexible, and surgeon-driven alternatives. Ongoing research will be essential to define the most effective and accessible strategies for optimizing outcomes in microsurgical breast reconstruction.

## Figures and Tables

**Table 1:** Demographic and clinical characteristics of patients.

Variable	Total (n = 208)	Pre-CTA (n = 32)	Post-CTA (n = 176)	*p*-Value
Age (years), mean ± SD	47.04 ± 8.55	42.35 ± 13.06	48.58 ± 5.32	<0.001
BMI (kg/m^2^), mean ± SD	26.21 ± 4.68	27.44 ± 6.43	26.03 ± 4.32	Ns
Hypertension, n (%)	16 (7.7%)	5 (15.6%)	11 (6.3%)	0.14
Diabetes, n (%)	23 (11.1%)	9 (28.1%)	14 (8.0%)	0.002
Unilateral reconstruction, n (%)	188 (90.4%)	28 (87.5%)	160 (90.9%)	Ns

Ns: not significant.

**Table 2:** Venous complications and surgical outcomes.

Complication	Pre-CTA (n = 32)	Post-CTA (n = 176)	*p*-Value
Postoperative venous congestion, n (%)	12 (37.5%)	14 (8.0%)	<0.001
Partial flap necrosis, n (%)	10 (31.2%)	13 (7.4%)	0.003
Total flap loss, n (%)	2 (6.3%)	1 (0.6%)	0.11
Re-exploration for second vein, n (%)	8 (25.0%)	12 (6.8%)	0.007

**Table 3:** Superdrainage and systemic complications.

Variable	Pre-CTA (n = 32)	Post-CTA (n = 176)
Superdrainage performed, n (%)	3 (9.4%)	72 (40.9%)
-Planned preoperatively, n (%)	N/A	65 (90.3%)
-Decided intraoperatively, n (%)	3 (100%)	7 (9.7%)
Superdrainage complications, n (%)	0 (0%)	0 (0%)
Thromboembolic events (DVT/PE), n	0	3

**Table 4:** Hospital stay duration.

Group	Mean Length of Stay ± SD (Days)	*p*-Value
Pre-CTA	9 ± 3	
Post-CTA	6 ± 2	<0.001

## Data Availability

No data collection forms, extracted data, analytic code, or other materials used in this review are publicly available but are available from the corresponding author upon reasonable request.

## Supplementary Materials

The following supporting information can be downloaded at https://www.mdpi.com/article/10.3390/jcm14175972/s1, Table S1: STROBE checklist.
