# Supplementary material for: Superficial vs. Deep Venous System in DIEP Flaps: Lessons from 25 Years of CTA-Guided Planning
Source: J Clin Med. 2025 Aug 24;14(17):5972. doi: 10.3390/jcm14175972 (PMC12429580; doi:10.3390/jcm14175972)
Supplement: Supplementary file 1 [file jcm-14-05972-s001.zip › jcm-3807186-supplementary.pdf]

Table S1. STROBE Checklist for Cohort Studies – DIEP Flap Reconstruction Study

Title and abstract

Indicate the study’s design with a commonly used term in the title or the abstract

✓Addressed in the manuscript.

Background/rationale

Explain the scientific background and rationale for the investigation being reported

✓Addressed in the manuscript.

Objectives

State specific objectives, including any prespecified hypotheses

✓Addressed in the manuscript.

Study design

Present key elements of study design early in the paper

✓Addressed in the manuscript.

Setting

Describe the setting, locations, and relevant dates, including periods of recruitment, exposure, follow-up, and data collection

✓Addressed in the manuscript.

Participants

Give the eligibility criteria, and the sources and methods of selection of participants. Describe methods of follow-up

✓Addressed in the manuscript.

Variables

Clearly define all outcomes, exposures, predictors, potential confounders, and effect modifiers

✓*Addressed in the manuscript.*

Data sources/measurement

For each variable of interest, give sources of data and details of methods of assessment

✓*Addressed in the manuscript.*

Bias

Describe any efforts to address potential sources of bias

✓*Addressed in the manuscript.*

Study size

Explain how the study size was arrived at

✓*Addressed in the manuscript.*

Quantitative variables

Explain how quantitative variables were handled in the analyses

✓*Addressed in the manuscript.*

Statistical methods

Describe all statistical methods, including those used to control for confounding

✓*Addressed in the manuscript.*

Participants (Results)

Report numbers of individuals at each stage of study

✓*Addressed in the manuscript.*

Descriptive data

Give characteristics of study participants and information on exposures and potential confounders

✓*Addressed in the manuscript.*

Outcome data

Report numbers of outcome events or summary measures over time

✓Addressed in the manuscript.

Main results

Give unadjusted estimates and, if applicable, confounder-adjusted estimates and their precision

✓Addressed in the manuscript.

Other analyses

Report other analyses done, e.g., subgroup analyses and interactions

✓Addressed in the manuscript.

Key results

Summarise key results with reference to study objectives

✓Addressed in the manuscript.

Limitations

Discuss limitations of the study, taking into account sources of potential bias or imprecision

✓Addressed in the manuscript.

Interpretation

Give a cautious overall interpretation of results considering objectives, limitations, and other relevant evidence

✓Addressed in the manuscript.

Generalisability

Discuss the generalisability (external validity) of the study results

✓Addressed in the manuscript.

Funding

Give the source of funding and the role of the funders for the present study

✓Addressed in the manuscript.
